# Clinical education during a crisis lived experiences of nursing students in Sweden during the COVID-19 pandemic

**DOI:** 10.1186/s12912-025-02714-9

**Published:** 2025-02-27

**Authors:** Elsa Nilsson, Lina Pousette, Lina Emmesjö, Mikaela Ridelberg

**Affiliations:** 1 The municipality of Jönköping, Jönköping, Sweden; 2 Skaraborgs sjukhus Skövde, Västra Götalandsregionen, Skövde, Sweden; 3 School of Health Sciences, University of Skövde, P.O. Box 408, Skövde, SE-541 28 Sweden; 4 School of Health and Welfare, Jönköping University, Jönköping, Sweden; 5 The city of Gothenburg, Gothenburg, Sweden

**Keywords:** Nursing education, COVID-19, Clinical skills, Phenomenology, Interviews

## Abstract

**Background:**

Nursing education entails extensive training across varying settings where nursing students can practice their theoretical knowledge and practical skills for their future profession. Skills in evidence-based practice are pivotal competences for nurses and need to evolve from novice to expert skills. During the COVID-19 pandemic, Sweden had a unique approach to restrictions. The conditions for nursing students to apply their practical skills changed, as the COVID-19 pandemic influenced nursing education. Previous studies lacked focus on the clinical and practical aspects of the nursing education during the COVID-19 pandemic. Such experiences can provide valuable knowledge for nursing education, especially in preparing for future crises as well as understanding the needs of the nurses who were educated during the pandemic. The aim of this study is therefore to highlight the essence and understanding of the experiences of nursing students undertaking nursing education in Sweden during the COVID-19 pandemic.

**Methods:**

A qualitative interview study of nine nursing students recruited through a convenience sample. The data was analyzed through a phenomenological hermeneutic approach, chosen for two of the opportunity to use the researcher’s preunderstanding. The result is presented in three main themes; Being disappointed yet accepting the situation, Feelings of uncertainty and the covid-19 pandemic provided new insights, the two latter are further divided into a total of eight subthemes.

**Results:**

The COVID-19 pandemic affected nursing students’ experience with clinical learning and contributed to their unique experiences. Compared to students who began their nursing education during the COVID-19 pandemic, those who started before the outbreak faced more significant adjustments in their learning. It was seen as positive for the student’s future profession that a great focus was placed on how to prevent the spread of infection. The students experienced loneliness in the lack of support from other students, and fear of infecting or being infected. Furthermore, there was a lack of clinical placement and training hours, leading to worries of not being prepared. Opinions differed between the students on whether the clinical knowledge they acquired during the training was sufficient. Whilst the vast majority wanted more time for practical elements and training, they emphasized that this was not solely caused by the impact of the COVID-19 pandemic but an effect of the design of the education.

**Conclusions:**

The study showed that, through the shared experiences of nursing students who studied during the COVID-19 pandemic, education and the acquisition of clinical skills were affected. The students experienced feelings of loneliness in their isolation and fear of infecting or being infected throughout their education. However, the majority found themselves with a sense of acceptance of the situation, while there were also experiences of pausing the education because of the pandemic. Being a part of the group of nursing students participating in education during the COVID-19 pandemic gave new insights into the nursing profession regarding hygiene routine and hindering factors in communication with patients. Nursing students who have gone through their education during the COVID-19 pandemic have unique experiences such as preparedness of coming pandemics and the importance of reflection surrounding clinical education that are worthy of sharing. These experiences could be helpful when developing nursing education in the future and preparing for possible future crisis situations.

## Background

In March 2020, the impact of the COVID-19 pandemic led to significant changes in nursing education globally. The conditions for nursing students to apply their practical skills changed [1,2] when health care organizations and universities had to make significant changes to their everyday work due to restrictions. Nursing education entails extensive training across varying settings and teaching and examination forms, where nursing students are able to practice their theoretical knowledge and practical skills for their future profession [3,4]. Skills in evidence-based practice, a pivotal competence for nurses [5,6], are needed for a nurse to evolve from a novice to an expert [7]. These crucial skills are developed in skill laboratories, in simulated scenarios [8,9], and during clinical practice. All these elements are needed to prepare nursing students for their future profession [10]. Nursing students may be especially vulnerable to the reduction of practical and clinical training due to the importance of practical skills in their future profession and the challenge they already faced in applying theoretical knowledge in a clinical setting [11]. In Sweden, skills laboratories were cancelled, digitalized or replaced with theoretical activities [12]. The practical training performed on campuses was limited by restrictions, which led to 50% of the students feeling less prepared when entering the clinical placements in Sweden and the United States [12,13]. Students who attended clinical placements during the COVID-19 pandemic perceived their level of competence as lower than students who had completed their placements before the pandemic according to research in the Czech Republic [14]. Furthermore, previous research in Europe and the Middle East has shown that nursing students experience stress, uncertainty, fear of infection, difficulty concentrating and learning, in addition to worrying about their competence in their future profession [1,15–17], death anxiety [18], social jet lag and depression [19]. However, nursing students also expressed that being a part of a historical event meant that they had to step up to a challenge [20].

Even prior to the COVID-19 pandemic, nursing students struggled to apply the knowledge they acquired at university to clinical practice^21,22,]^ where reflection is required to integrate theory and practice [9,11]. Nurses’ clinical knowledge, development and decision making were identified and described in the model from novice to expert [7]. The model expresses how novices lack experience and therefore abide by rules and guidelines, the advanced beginner nurse demonstrates development of skills in performing nursing tasks but is still in need of guidance, a proficient nurse has a holistic understanding and the expert nurse abide by intuition to understand the situation and quickly recognize patterns based on extensive experience [7]. During COVID-19 expert nurses became novices when needed to change work tasks due to the pandemic [23], who were also supervisors of the nursing students on clinical placements.

Sweden had a different approach to restrictions [24,25], focusing on recommended restrictions [25,26]. In contrast to restrictions recommended globally [24,25]mandated interventions restricting personal freedom were not mandatory in Sweden. Nursing students who have received their education during the COVID-19 pandemic have unique experiences in clinical education that are worth exploring. The previous studies found lacked focus on both the clinical and practical aspects of the nursing profession in the Swedish during setting during COVID-19. How students experienced their educations clinical setting during COVID-19 in Sweden could be helpful when understanding the needs of the nurses educated during the COVID-19 pandemic, as well as when developing nursing education in the future.

## Methods

### Aim

The aim of this study was to highlight the essence and understanding of the experiences of nursing students undertaking nursing education during the COVID-19 pandemic.

### Design

A qualitative interview study with a phenomenological hermeneutic approach was conducted to describe nursing students’ experiences with clinical learning and teaching during the COVID-19 pandemic. The phenomenological hermeneutic approach was deemed relevant since it is appropriate for capturing lived experiences. Furthermore, the approach allowed for the use of the researchers’ preunderstanding, which was appropriate since two of the authors had been nursing students during the pandemic.

### Study setting

Swedish nursing education is a three-year-long baccalaureate education programme that, after completion, provides the opportunity to become a registered nurse. The Higher Education Ordinance [27] regulates Swedish nursing education and determines the goals that the student must fulfill to obtain his or her degree. Education is also regulated at an international level via directives from the European Union (EU) and the European Economic Area. These directives require that half of the nursing education consist of clinical training. Clinical training must take place either in a hospital or in other health care facilities where patient care is carried out under the supervision of a registered nurse [28]. Initially during the COVID-19 pandemic, universities providing nursing education followed the recommended restrictions and moved education from campus to digital. Skills labs were closed, where skills training and simulations were cancelled. Clinical placements were cancelled in several parts of the country, but at some universities it was only applied to terms 1–4. As the pandemic progressed, protective gear was worn as the skills labs opened with restrictions, limiting the number of students and times which the skills lab could be used.

### Participants

Study participants were recruited through a convenience sample [29], to participate in a semi structured interview about their experiences with clinical learning and teaching during the COVID-19 pandemic. To facilitate recruitment, the authors contacted one student union, one accident and emergency clinic and a Facebook group, which included nursing students and newly graduated nurses in Sweden. Informed consent was given by the administrators of the Facebook group to the participants. This recruitment approach was intended to broaden the inclusion of participants from several universities across Sweden. A post was made in the Facebook group, which included information about the study. The information contained an information letter that included the aim, method, responsible researcher and responsible organization. Members of the group sent their email addresses to the researchers, who then sent out an information letter to further inform them about the study. To participate in the study, individuals had to be enrolled in nursing education for at least two reasons at a Swedish university during the COVID-19 restrictions. Nine nursing students or newly graduated nurses participated in the data collection; five from the Facebook group, one from a student union and one from an accident and emergency clinic. The two participants from the student union and emergency clinic were contacted via direct message and were associated with one of the authors. Two pilot interviews were conducted with two of the authors’ (E.N. & L.P.) classmates. These two interviews were later included in the Results section, as they provided valuable insights. The students who participated were either mid-semesters or early-semesters or had not even started study when the COVID-19 pandemic broke out. The participants’ age and sex were not requested, as such information was not relevant to the study aim.

### Data collection

The data were collected through semi structured interviews, which were conducted through video calls. Digital interviews enhance the opportunity for long-distance participation and are cost effective [30,31], and are compatible to in-person interviews in terms of quality of data [32]. Technical difficulties may arise which may influence the quality of the interview during digital interviews [33]. No such issues occurred, and the audio and video were experienced as of high quality. The interviews were recorded, lasted between 22 and 38 min and were transcribed verbatim. Nonverbal expression was noted during the interviews. The first interview question was “What are your experiences with clinical education/teaching during the COVID-19 pandemic?”. Probing questions were asked to delve deeper into their experiences, such as “Can you elaborate?”, “What did you feel then?” and “Do you have an example?”.

### Data analysis

The data were analyzed through a phenomenological hermeneutic approach [34–36]. The approach was considered relevant since the aim was to highlight the essence of the participants’ experience of the phenomenon. Through a phenomenological approach, the participants’ lived experience of a phenomenon can be obtained through directing the participants’ focus to the phenomenon. Phenomenological analysis becomes possible when the transcribed material is processed and organized through the researcher’s preunderstanding of the phenomenon. According to philosophical hermeneutics by Gadamer [37], understanding a phenomenon necessitates drawing on the authors’ preunderstanding and its historical context which were done in the analysis of this manuscript. Gadamer’s concept horizon describe what is possible to understand, and the limits for understanding, where the use of one’s preunderstanding is expressed to be what makes science secure [37]. For the interpretation of the data to become trustworthy, an openness is required surrounding the researchers’ preunderstanding of the phenomenon. Two of the researchers (E.N. & L.P.) conducted their first year of nursing education with COVID-19 restrictions. The other two researchers were actively teaching in nursing education during the COVID-19 pandemic. These experiences affected the researchers’ preunderstanding of the phenomenon, which was used to deepen the understanding. The preunderstanding of the researchers may however have influenced the data analysis by being influenced by the researches’ bias. Continuous discussions within the research group as well as with a reference group of other nursing students were conducted throughout the analysis process to avoid bias. The analysis included three stages: naïve understanding, structural analysis and conclusive understanding. The transcribed interviews were therefore read several times, after which the researchers wrote down their naïve understanding of the phenomenon. Second, the transcribed data were structured into meaning units which answered the study aim. The meaning units were copied out of the document, and then summarized through condensing them while keeping their essence. The condensed meaning units were sorted based on similarities of the contents. The sorting process were discussed among the research team and were then divided into subthemes and themes. The final analysis step included a reflection on the meaning units in relation to the preunderstanding of the researchers for a deeper understanding of the findings.

### Rigor

The rigor of qualitative research is determined by the trustworthiness of the confidence in the data and the findings. The four criteria—credibility, transferability, dependability and confirmability [38]—will be used to discuss the trustworthiness of the results. Credibility evaluates integrity, quality and confidence in findings based on how the research is conducted and reported [39]. The informants were chosen because they had experience with the phenomenon. In qualitative research, dependability is described as a criterion for evaluating integrity. It refers to the stability of data across different contexts and over time [39]. The informants interviewed were students who had undergone two periods of the nursing program. during the COVID-19 pandemic. Since the students’ experiences covered an extended period of time, this contributes to the dependability of the findings. Transferability refers to how the results can be transferred to other settings or groups [38]. The method of recruitment provided a geographic spread where students from six different universities participated. Confirmability is a criterion for integrity in a qualitative study. The criterion is established when credibility, dependability and transferability are achieved [38]. The description of the participants, recruitment, data collection and analysis can hopefully enable the reader to consider the confirmability of the research findings.

### Ethical considerations

The study was conducted in accordance with the four ethical principles of the Declaration of Helsinki – respect for autonomy, beneficence, nonmaleficence, and justice [40]. The study was designed, planned, and performed as per Swedish law^41,]^ which states that ethical approval is not needed when people are invited to participate voluntarily in interviews. In this case, the participants were not considered vulnerable since they were interviewed about their nursing education experiences by other nursing students. Two of the participants were classmates of two of the researchers, which could be an ethical issue since the participants may have felt pressured to participate. The two participants did not have private association or collaborations in school with any of the authors. The two participants, along with all the other participants were provided with written information about the study aim was provided through the information letter, and at the start of the interview, confidentiality and the voluntary nature of participation were emphasized, as was the option to withdraw their participation at any time. The authors obtained informed consent to participate and publish, both verbally and with a signed form.

## Results

An overview of the three themes and eight subthemes that emerged from the data analysis is shown in Table 1. Against quotations provided in the text below, participants are coded by numbers.

**Table 1:** Structure of findings in themes and sub-themes

Theme	Subtheme
Being disappointed yet accepting the situation.	
Feelings of uncertainty.	Uncertainty due to changing information. Feelings of loneliness due to distancing. Fear of being infected.
The covid-19 pandemic provided new insights.	Unique knowledge from being a student during the pandemic. Reflection is important for learning. The importance of allocating time based on needs. A desire for more skills training. The theoretical knowledge and clinical skills belong together.

### Being disappointed yet accepting the situation

When nursing education and society changed overnight due to the COVID-19 pandemic, students had to accept the new situation they were facing. The students expressed feelings of disappointment but also acceptance related to the situation. Restrictions and distance education have reshaped and affected everyday life in several ways. Several students described how these changes gave them an understanding of their future profession. Understanding served as a bridge to acceptance and to face the difficulties brought about by the changes. The students needed to accept the conditions that prevailed both at the Nursing Skills Laboratory (NSL) and at the clinical placement.

“*When I see a patient with COVID-19*,* I have not thought much about it. They need care just as much. Additionally*,* I will meet these patients later when I will work by myself later*.” – Interview 5.

Universities in Sweden offered different solutions to ensure clinical knowledge and skills. Several students described how it is difficult to compare anything other than their own experience. Despite students experiencing feelings of injustice and questioning how the universities have chosen to plan their education, most have accepted their situation.

*“… it felt a bit strange*,* but still*,* or at least I accepted the situation. I knew that it’s not optimal for anyone.” - Interview 7*.

There has also been disappointment in the outcome and how it has affected the students’ education and the conditions for clinical learning. Students who began their studies before the outbreak of the COVID-19 pandemic faced greater adjustment. Most students studied a programme that was designed to be campus-based, which led to disappointment in the lack of campus-based education. The cancellation or replacement of clinical skills classes with theory classes left students feeling disappointed. Disappointment stemmed from the uncertainty of whether there would be an opportunity to practice clinical classes in the future or whether they would need to rely on theoretical knowledge in their working life. College and university students clearly had different approaches to completing their education during the COVID-19 pandemic. The students reference hearing about others’ experiences. One student specifically mentioned how the changes in education made them take a break from their studies.

“*I quit. Or… I took a break because I felt that if I continue*,* I will not have the same knowledge and experience as my future colleagues. Now that covid has decreased*,* I have continued my studies*.” – Interview 4.

### Feelings of uncertainty

The theme of feelings of uncertainty is divided into subthemes. An indefinite flow of information combined with feelings of loneliness as a result of distancing and fear of being infected impacted the students’ education. The students stated that information from the authorities and the university was perceived as unclear, which brought uncertainty regarding what applied. Distance education and a lack of connections with classmates produced feelings of insecurity among the students themselves. The risk of being infected by COVID-19 also led to fear and uncertainty when patients and classmates were met.

#### Uncertainty due to changing information

The students described feelings of uncertainty during their clinical training due to the pandemic. The students who were in a clinical placement when the COVID-19 pandemic broke out in Sweden described how they were sent home, leaving them feeling uncertain about what would happen next.

*“Yes*,* I think it was two or three days when the university contacted our supervisors. They said*,* ‘Send the students home; we do not know what to do*,*’ and added*,* ‘However*,* they should not be in the hospital.’ Then*,* there was silence. I think we were at home for a few days*,* and we did not do anything.”* – Interview 7.

During the pandemic, recommendations and restrictions changed continuously, which meant that routines involving protective equipment were constantly changing. The students described that it was difficult to keep up to date with what was actually applied, both outside the clinic and at the NSL. The indefinite flow of information from both universities and health care institutions is described as affecting education. Students reported feeling troubled by uncertainty regarding mask wearing and university closures.

“…*There was uncertainty about what kind of restrictions applied when we were at school and not at school*,* “when should one use a face mask and when it not? When was the NSL open? And when NSL not open? what were we allowed to do and what were we not allowed to do?“. I thought that was a bit difficult when you didn’t know what was…” – Interview 9*.

Moreover, the lack of clear communication required them to check with each other for updates. Restrictions at the NSL meant that it became difficult for the students to access complete skills training. This included both mandatory scheduled training and clinical skills training in the NSL.

*“…yeah okay*,* the university expects us to know this*, but we cannot practice anywhere. What the f*ck are they thinking?” - Interview 7.

#### Feelings of loneliness as a result of distance

Studying during the COVID-19 pandemic was characterized by a feeling of loneliness. Distancing measures during the pandemic hindered opportunities to connect with classmates, leaving students lacking readily available support and a lack of context. Forming relationships with classmates is described as facilitating learning in several ways: exchanging experiences, discussing assignments, coordinating bookings for the NSL and feeling more comfortable asking questions. A lack of context was described as affecting self-perception. A feeling of inadequacy and uncertainty arose because there were no classmates talking to about one’s thoughts or whether one’s knowledge was sufficient.

*“It is been very lonely. The time as a nursing student during the pandemic was characterized by uncertainty and feelings of inadequacy.” –* Interview 3.

Nursing students acknowledged that when the restrictions subsided, teaching became less distant. The opportunity to get to know each other made the studies more enjoyable, and the feeling of loneliness decreased.

#### Fear of being infected

Some of the students described their feelings of fear as being present in several different situations. Students highlighted how they kept themselves updated on the spread of the virus, the death toll, and how that affected them. One student described the fear of being infected because it was not possible to know how other students followed the pandemic restrictions. The fear of not being able to complete their education caused frustration and insecurity among the students. Clinical placements, which are a significant part of the nursing programme, were located in health care units that were affected by the pandemic. There was an obvious risk to health workers before vaccines and adequate protective equipment were available. This significantly affected the students in relation to the importance of education.

*“We’re coming here with our lives at stake*,* but we did not get paid because we were studying. This was a topic of conversation among many students. They thought it was difficult because they did not want to get sick*,* of course. Was it worth it?” –* Interview 7.

### The COVID-19 pandemic provided new insights

The theme of the COVID-19 pandemic provided new insights and was divided into several subthemes: unique knowledge from being a student during the pandemic, reflection important for learning, the importance of allocating time based on need, a desire for more skills training, and theoretical knowledge and clinical skills. During the COVID-19 pandemic, nursing students had to adjust in many ways, which gave them unique knowledge. The importance of reflecting to process new knowledge was further emphasized when time constraints limited the possibility of this. Time could be perceived as short, and the students came to the realization that it was best to allocate it to the most necessary. A desire for more skills training emerged as a common theme among the students, regardless of how prepared they felt for their future profession. The fact that theoretical teaching took place remotely was experienced differently by the students, and this negatively affected their clinical knowledge.

#### Unique knowledge from being a student during the pandemic

Being a student during the COVID-19 pandemic was experienced as a bonus in terms of knowledge, knowledge of health care hygiene and infection control, and understanding of nursing during a pandemic. Unique knowledge emerges when students describe their experiences. The students explained that the increased focus on routines for hygiene and infection control has been something that has affected not only their own practices as health care professionals but also society. The students expressed that it has given them security in how infections should be dealt with in their future profession. COVID-19 is also described as having increased accuracy and given a holistic perspective on how materials should be provided and handled in a clinical environment.

*“There is nothing to say there will not be new pandemics in the future. We’ve been prepared and understood.” –* Interview 1.

Moreover, wearing a mask and visor also strengthened the ability to communicate. When facial expressions cannot be relied upon, other nonverbal expressions of body language, tone of voice and pauses develop. Some students feel secure in the clinical knowledge they have acquired and believe that their work in health care may have contributed to this. Some of those who had time to work as nurses at the time of the interview expressed that the clinical knowledge provided by the university lacked sufficient or practical application compared to the realities encountered in the clinic itself.

#### Reflection is important for learning

The need to reflect in different contexts was illustrated by the students. The students identified a lack of reflection opportunities outside of the classroom with their classmates, during lessons and during clinical placements with supervisors. Reflection was described as facilitating learning, creating space for conversations about experiences and feelings and contributing to security.

*“… I feel that the pandemic affected my clinical learning on two levels. One is the clinical part*,* and the other is that there was a lack of exchange with other students. I think that the second one was actually much*,* much more important in some way for me.” –* Interview 3.

During clinical placement, supervision is experienced as a central part of clinical education as a whole. Reflection was described as important for learning, and being able to reflect on one’s experiences during clinical placement was something that was not always possible during the COVID-19 pandemic due to strain on the clinics.

*“… it also made me feel that when I maybe did not have the theoretical knowledge to be in the hospital*,* they (supervisors) could not support me in that part either when I was in the hospital because they did not have time to talk. It was very stressful.” –* Interview 6.

A shortage of time resulted in no time for reflection, which affected the overall experience of clinical placement for several of the students. The clinical placement was experienced as chaotic due to the prevailing workload. Sick leave and staff shortages led to a lack of continuity in supervision.

#### Importance of allocating time based on needs

Several perspectives on time can be found, and making time and catching up were recurring aspects. The responsibility for making time was described as being down to the individual student, the university’s structure and the clinical placement. A shortage of time was explained as a result of restrictions, stress and heavy workloads as well as time spent on other assignments.

*“…there was a lot of time and energy that went into just making things work.” -* Interview 1.

Students, supervisors and teachers needed to reallocate and prioritize time. These groups held different perspectives on what was deemed most valuable and important. The prioritization of time could be seen in one student’s reflection on how taking the time to visit drop-in practices became difficult.

*“It’s easy to be lazy and stay at home instead of taking half an hour and being at the drop-in clinical practice.” –* Interview 9.

The fact that the restrictions led to a limited number of groups at the various skills training sessions was described as having a negative effect. Smaller groups made it more difficult to book a time slot at NSL. This created frustration for the students. The reduced number of students in the groups was positive, as the teachers had the opportunity to spend more time supervising the students in the skills exercises at NSL. It was then perceived as more important and profitable than when the skills training was held in larger groups.

#### A desire for more skills training

The students had varying experience with whether they felt sufficiently prepared in terms of their clinical skills. Different institutions of higher education had to change their study plans to varying degrees. The plan was sometimes not perceived as being sustainable. Students feel that the practical part of the education, despite limitations, provided a good foundation for their future profession. All the students described a desire for more skills training in preparation for their nursing profession but stated that this desire was not related to the COVID-19 pandemic.

*“I would like to have more clinical training. Not that*,* COVID-19 took that away*,* but I would like to have more clinical training*,* and I would like to have more access to NSL. I think everyone would benefit from that.” –* Interview 2.

#### The theoretical knowledge and clinical skills belong together

Nursing education in Sweden includes both clinical and theoretical goals. Students describe this approach as fostering a holistic understanding of the profession. The fact that the pandemic created other conditions for students to acquire theoretical knowledge through remote studies has thus also affected how they acquired practical knowledge.

The wording

*“Both the clinical and theoretical parts were affected; for example*,* sitting at home by yourself also affected the clinical part.”* Interview 3.

Remote studies have been conducted differently. Some have appreciated the freedom and opportunities to manage their own time and the convenience of accessing digital study materials.

*“Yes*,* precisely because the theoretical goes into the clinical.”* - Interview 6 responding on how digital studies affected the clinical aspects of nursing.

The recorded lectures were perceived as helpful for learning, and this had a positive impact on clinical knowledge. Distance education and learning have also been experienced as troublesome and difficult to relate to.

## Discussion

The results showed that students’ experience with clinical education during the COVID-19 pandemic provided unique experiences. The experience was considered positive by the students, which has been expressed in previous research in which both clinical teachers and students experienced an opportunity to learn from the crisis that arose during the COVID-19 pandemic [12,42]. Below is a discussion of the influence of COVID-19 and how it may affect students in their future profession.

The clinical placement during the pandemic was expressed by the students in this study to have been chaotic and strained, which Engqvist Boman et al. [43] also emphasized in their study that the students experienced clinical placement as chaotic but also of great importance in consolidating clinical skills, leading to increased self-confidence. The lack of support from supervisors in this study was seen as a hindering factor in acquiring new knowledge and made the students doubt whether the existing theoretical knowledge was sufficient. Feeling comfortable at clinical placement facilitated the learning process during the COVID-19 pandemic and helped students acquire knowledge in nursing and face difficult situations [44]. The students in the present study expressed that the clinical placement could be chaotic and that continuity from supervisors was lacking and affected the overall experience. A lack of continuity was perceived as hindering the experience of clinical placement. This could indicate that the students lacked support from supervisors during their clinical placement during the pandemic and therefore had difficulties accommodating clinical skills. This could affect the acquisition of professional skills in relation to Benner’s [7] model. This study, along with previous research [43], showed that being a nursing student during a pandemic can equip them with an increased level of preparedness and understanding when facing new pandemics and crises.

From the results of this study, it appears that the students experienced uncertainty due to unclear information from the universities regarding which restrictions and routines were current at campus and out in the clinics. Uncertainty is also found to be an emotional aspect in which loneliness and fear are prominent. This has been observed in previous research [43], which describes how students experienced insecurity in relation to themselves, their relatives, the health care staff, patients, the clinic, and society at large. Kaveh et al. [42] also found fear concerning the risk of becoming infected or infecting others. This fear was described to be intensified by a lack of protective materials and an unsustainable workload, which was also observed in this study. Taken together, these findings suggest that nursing students experienced significant disruptions in learning due to COVID-19 restrictions, which exacerbated anxiety and worry. This was also expressed by the students in the present study. It is possible to state that feelings of loneliness and fear originate from the individual and are influenced by many other external factors that were not further examined in this study.

The results showed that some of the students felt that their clinical skills had been affected. Some decided to take a break from studying because of the perceived low quality of education during the pandemic. There was a fear of not having the competence required at the end of the education. Higher Education Authority [12] noted that when skills training at universities was limited by the COVID-19 restrictions, students felt less prepared for practice at the NSL. Students described that the reduced number of clinical training hours during the COVID-19 pandemic contributed to reduced clinical skill readiness [13]. Kaveh et al. [42] reported that the clinical training of nursing students has been affected in such a way that learning opportunities have been lost due to the pandemic. It also identifies a deficiency in the programme’s ability to provide adequate opportunities for students to develop skills. The students in this study expressed mixed feelings about whether they felt confident in the clinical skills they acquired from the training. Those who graduated and started working felt that they lacked sufficient preparation for the profession and work outside the clinic. Ridelberg et al. [45] suggest that insufficient skills of nurses are a barrier to increased patient safety. Being a newly graduated nurse was also referred to as a barrier. It is thus possible to conclude that patient safety could suffer because of the lack of security and preparation for students in their clinical skills. A lack of security can also be seen as a hindering element for nurses to move through the Benner’s [7] model of professional competence. The impact of the pandemic on students varied depending on their stage in the programme since restrictions and recommendations were constantly changing. Luo et al. [43] argue that nursing students studying in their final year when the COVID-19 pandemic broke out quickly took on the role of professional nurses. This differs from the students’ experiences in this study, which meant that it took time before they felt safe. One could reason why the impact on the students was dependent on the stage they were at in their education when the transition induced by the pandemic took place.

The students felt that the pandemic and its restrictions limited the possibility of reflection with fellow students, which was considered negative. Reflection was described as important for accommodating knowledge of clinical skills by helping students share experiences and learn from each other, which aligns with the findings of previous research^43,]^ which highlights how students experience reflection as an important part of the learning process. This study showed that opportunities for reflection were not always available due to the pandemic. Gustavsson and Berglund [46] describe reflective education in groups as a concept that provides opportunities for different types of knowledge to be developed where joint learning occurs. Sundler et al. [47] reported that feedback and reflections were considered very significant for students’ learning and self-esteem during clinical placement. A lack of reflection may therefore have affected the students’ opportunities to take advantage of clinical skills in a favorable way, thereby also limiting the possibility of acquiring professional skills according to the Benner’s [7] model. Luo et al. [48] highlights how nursing students emphasized the importance of support from each other as a coping mechanism to meet the challenges of changes in the clinic during the COVID-19 pandemic. It can therefore be considered important to consider support from both supervisors and fellow students when students face challenges during their clinical placement.

Several students felt that education during the pandemic focused heavily on hygiene routines and their significance in preventing the spread of infection. A similar study also noted a focus on hygiene^43,]^ which highlights how students were attentive to whether others followed hygiene routines. It can therefore be assumed that nursing students who studied during the pandemic have a deeper understanding of hygiene routines and their meaning, which is in accordance with the International Council of Nurses [49] code of ethics and the Swedish Nursing Association [1] competency description.

During the COVID-19 pandemic, students needed to explore new ways to communicate with patients. This was also described in a study by Russo et al. [44]. Engqvist Boman et al. [43] highlights how nurses’ educational knowledge is strengthened when the connection between theoretical knowledge and patients’ needs for verbal and nonverbal communication is clarified. Furthermore, the students also drew attention to masks and visors as barriers to communication, and some patients became fearful and anxious about the protective equipment. Saunders et al. [50] concluded that face coverings had a negative impact on hearing, understanding, feelings of connection and engagement in terms of communication. Face cover also impacted content, interpersonal connectedness, and the willingness to engage in conversation.

The Benner [7] model of nursing competence outlines the development of a nurse’s professional skills through intervention and experience. This explains why competence development is not a linear phenomenon since the individual moves both forward and backward in relation to different skills. The Benner [7] model stresses that even an experienced nurse can become a novice in an unfamiliar situation. During the COVID-19 pandemic, it can be assumed that many experienced nurses and supervisors became beginners in relation to the prevailing situation and that students and supervisors learned jointly to master the situation. The reported challenges and uncertainty surrounding supervision during clinical placement likely hindered students’ development of professional skills, as outlined in the Benner [7] model.

The results also showed that the students wanted more skills training and expressed this as something independent of the COVID-19 pandemic. On 26th January 2023, the Swedish government issued an ordinance amending the Higher Education Ordinance [27,51], where it is stated that nursing education must include at least 2300 h of clinical training. In accordance with the directives of the EU/EEA^28^, which state that nursing education must consist of 50% clinical training, this change comes with risks. Without an extension of the nursing programme, this means that other elements of the programme will have to be reduced, thus likely affecting the entirety of the content. Additional clinical training puts greater strain on the clinic. It also appears that there is a need to further define clinical education since clinical learning does not only take place at the clinic. This means that students’ desire for more clinical training will become reality, which may help them acquire knowledge and excel in clinical knowledge in relation to the Benner [7] model.

### Limitations and strengths

An impediment of the study was how when choosing informants, there was a desire to avoid interviewing acquaintances, to avoid the authors’ relationships’ affecting how the participants responded. However, acquaintances were interviewed in the pilot interviews because they were accessible to the researchers in the early research process. Due to the limited experience of the authors who conducted the interviews, the first two interviews were pilot interviews with classmates. Interviewing classmates in the study could be seen as a limitation, as the authors of the study can also have an impact on how classmates responded. The impediment were overcome by assuring that the participants in the pilot interviews did not have private association or collaborations in school with any of the authors. Furthermore, there were a discussion prior to, as well as after the interviews with the other researchers. The pilot interviews were deemed to enrich the understanding of the phenomenon and excluding them would have limited the results. Including these patients was therefore considered beneficial to the study. Furthermore, the authors’ desire to try and establish which interview questions were suitable for investigating the phenomenon, where carrying out the pilot interviews strengthened credibility. The geographical spread gives the study a nuanced result, which is a strength that increases the transferability of the result. The possibility for a reader to determine if the findings are transferable is made possible by the thorough description by participants, the research context and the analysis process in the methods section. However, the transferability of the findings to other contexts may be difficult for the reader to judge [38].

## Conclusion

The study showed that nursing students who studied in Sweden during the COVID-19 pandemic shared the experience that the pandemic has affected education and the acquisition of clinical skills. The students experienced feelings of loneliness in their isolation and fear of infecting or being infected, but they also found themselves feeling a sense of acceptance as well as gaining new insights of their profession. The pandemic’s impact on education thus elicited both positive and negative experiences. Preparation and readiness within the clinical field of nursing for their future profession is something all the nursing students expressed to want more of, even when they felt confident in their skills. It can be concluded, therefore, that there is a wish to always feel confident in clinical skills as a nursing student. Unique experiences of being educated during a global pandemic provided deeper knowledge on the importance of hygiene and preventive work, were mentioned as positive outcomes. A finding, which can also be found in other studies, is that students experienced a need for support from both classmates and supervisors to accommodate clinical knowledge in their education, which was lacking during the pandemic. Support was shown to be significant for learning, and it also gave participants a sense of belonging. Since the COVID-19 pandemic has brought uncertainty to several aspects, the importance of reflection has been further acknowledged. Clinical learning is central to nursing education; therefore, how it is experienced has great importance from an overall perspective. Clinical knowledge is both clinical skills and theoretical knowledge, both significant to when growing into an expert nurse.

### Clinical implications and proposals for the development of the subject

The results showed that education was affected by the pandemic, and in some cases, the quality of education was perceived as unsustainable for the students. It is therefore important that institutions of higher education that educate nurses assimilate the results to further develop the structure of education in a sustainable way. The COVID-19 pandemic and its adjustment had an impact on the students’ education experience. Students’ experiences offer valuable insights that can help to inform practices to better prepare students for similar situations in the future. In this study, reflection has been shown to have great significance for students’ experience with the profession. It brings a sense of security and learning ability, which should be considered by both universities and clinics. Whether the clinical competence of nurses, who studied during the COVID-19 pandemic, has been affected has not been definitively answered, but clinics should take this aspect into account. It can be argued that patient safety may be affected by shortcomings due to a feeling of insecurity and lack of confidence in their role as a nurse. This further contributes to the need for understanding and adjustment in the clinic. Therefore, the results of this study should be of interest to clinicians. Suggestions for further studies could include investigating more closely how the phenomena of fear and loneliness affect studies and further investigating how patient safety has decreased during the COVID-19 pandemic in relation to the presence of nursing students at clinics. During the execution of this study and translation of the text from Swedish to English, further implications emerged for defining and examining the concept of clinical learning and the different aspects of the phenomenon. A concept analysis of clinical learning could therefore be valuable. The study explored the difference between gaining knowledge within the theoretical field of practical skills and performing actual practical clinical skills. This study has raised the questions of where knowledge and skills meet and how they are defined. This topic should be studied further.

## Data Availability

The datasets analyzed during the current study are not publicly available due to ethical principles, but data not comprising confidential information are available from the corresponding author upon reasonable request.
